# Multi-objective Big Bang Big Crunch framework for reliable rice disease and variety classification with conditional calibration

**DOI:** 10.1371/journal.pone.0340807

**Published:** 2026-03-20

**Authors:** Chatter Singh, Amar Singh, Sahraoui Dhelim

**Affiliations:** 1 School of Computer Applications, Lovely Professional University, Phagwara, Punjab, India; 2 School of Computing, Dublin City University, Dublin, Ireland; Kafkas University: Kafkas Universitesi, TÜRKIYE

## Abstract

Deploying rice disease detectors in the field remains challenging because models that are accurate in the lab are often poorly calibrated and provide limited uncertainty estimates, raising the risk of costly misclassification. This paper proposes a multi-objective Big-Bang Big-Crunch (MO-BBBC) framework that jointly performs disease detection and variety classification while optimizing six deployment-oriented criteria: classification error, calibration quality, uncertainty estimation, model size, inference latency, and energy consumption. The proposed framework presents *conditional temperature scaling*, an adaptive scheme that mitigates over-calibration and preserves reliability. The framework is implemented in Python on a lightweight two-headed classifier and evaluated on the Paddy Doctor dataset, MO-BBBC base framework achieves 90.6% disease accuracy and 97.9% variety accuracy; improves calibration to AECE=0.0138  (≈70 % better than strong post-hoc baselines); achieves micro-AUC of 0.994/0.999 and micro-AP of 0.961/0.994 (disease/variety); delivers robust OOD detection (AUROC = 0.887/0.886); and supports real-time inference at ≈0.48  ms and 0.47  ms per 64-sample batch on CPU/GPU with Monte Carlo Dropout uncertainty. The resulting Pareto set enables practitioners to trade accuracy for efficiency and reliability, narrowing the gap between prototype validation and field deployment in precision agriculture.

## 1 Introduction

In real-world agriculture, it is as important to know how *confident* a disease detector is as it is to know *what* it predicts. Farmers must be able to interpret a statement such as “95% confidence of rice blast” as a calibrated probability, i.e., among predictions issued at 0.95 confidence, approximately 95% should be correct or they may underreact to genuine infections [1,2]. However, most plant disease recognition systems prioritize accuracy alone and produce poorly calibrated confidence estimates that misrepresent true disease risk [3,4]. This lack of calibration and uncertainty reporting undermines transparency and hinders adoption in agriculture [5–8].

To address this gap, this paper proposes a multi-objective Big Bang–Big Crunch (MO-BBBC) framework that optimizes six deployment critical objectives: (1) classification accuracy to support crop-protection decisions; (2) probability calibration so that predicted confidences reflect empirical frequencies; (3) uncertainty estimation to enable selective prediction and human escalation; (4) model compactness for edge deployment; (5) inference latency for real-time field use; and (6) energy usage for battery-constrained operation. This joint optimization mitigates the accuracy–reliability trade-offs that limit current agricultural AI systems. We further propose *conditional temperature scaling*, which applies calibration only when validation performance improves, avoiding overcalibration and preserving reliability. In addition, this research work combines Monte Carlo Dropout with predictive entropy and Bayesian Active Learning by Disagreement (BALD) to obtain uncertainty-aware predictions and strong out-of-distribution (OOD) indicators. Together, these components provide a practical and dependable solution for rice disease and variety identification.

Research in agricultural computer vision has advanced along several fronts, including multi-task learning for simultaneous disease and variety classification [1,3], compact architectures for mobile and edge devices [9,10], and uncertainty estimation [11,12]. Despite this progress, prior work remains fragmented: a framework that integrates calibration-aware training, deployment-conditioned model selection, and multi-objective efficiency optimization is still lacking [13,14]. Meanwhile, the broader machine learning literature offers principled calibration and risk-control methods (e.g., temperature scaling and selective prediction) [15–17], but their adaptation to resource-constrained agricultural settings with explicit reliability guarantees is underexplored.

**Contributions.** We advance trustworthy agricultural AI through four innovations:

(i) **Multi-objective deployment optimization:** a BBBC-based framework that jointly trades off classification error, calibration quality, uncertainty informativeness, model size, latency, and energy usage, yielding Pareto-optimal solutions with principled knee-point selection.
(ii) **Conditional calibration:** an adaptive temperature scaling scheme that is applied only when it improves validation criteria, preventing overcalibration while preserving multi-task performance across disease and variety heads.
(iii) **Uncertainty-aware inference:** integration of Monte Carlo Dropout with predictive entropy and BALD for selective prediction and OOD detection, communicating uncertainty explicitly to end users.
(iv) **Deployment-centered evaluation:** comprehensive validation with leakage-guarded splits, ablations, efficiency profiling, and comparisons against matched-budget search baselines (Random, TPE, NSGA-II).

In choosing the six optimization objectives, we explicitly align the search space with real agronomic decision pressures. Classification error directly controls the expected frequency of wrong recommendations, which translates into yield loss or unnecessary chemical usage. Calibration quality determines whether a nominal “95% confidence” score can be trusted when setting action thresholds or escalation rules. Uncertainty quality, summarized via E-AURC and NLL, governs how effectively the model can support selective prediction and abstention, allowing field users to defer uncertain cases rather than act blindly. Model size, latency, and the energy proxy together reflect deployment feasibility on edge devices where memory, responsiveness, and battery life are constrained. Optimizing these six objectives jointly, therefore operationalizes the trade-offs that agronomists, extension agents, and farmers face when choosing between more accurate but heavier models and slightly less accurate but leaner and more interpretable alternatives.

We validate the framework on the *Paddy Doctor* dataset [18] using a lightweight two-head classifier. The results show strong accuracy together with substantially improved calibration, uncertainty estimation, and efficiency, while meeting real-time and low-energy constraints. By narrowing the gap between laboratory performance and field deployment, this work contributes to trustworthy AI for precision agriculture.

The remainder of the paper presents related work (Sect 2), proposed methodology (Sect 3), presents experimental results (Sect 4), discusses deployment implications (Sect 5), outlines limitations and future work (Sect 6), and concludes (Sect 7).

## 2 Related work

This section places our contribution at the intersection of (i) calibration and uncertainty quantification (UQ) for decision support in agriculture, (ii) selective prediction and post-hoc calibration, (iii) multi-task learning (MTL) for plant health, (iv) resource-efficient models and edge-deployment, (v) multi-objective hyperparameter optimization (HPO) and deployment conditioned selection, (vi) out-of-distribution (OOD) awareness and dataset shift, and (vii) rice-specific and field-oriented systems. We focus on where previous work emphasizes accuracy or efficiency as opposed to being under-specified in terms of reliability (calibration and UQ) for the field.

### 2.1 Calibration and uncertainty in vision for agro-ecosystems

Probability calibration is now at the center of risk-sensitive decision-making because confidence scores are often a misrepresentation of empirical correctness, in particular, with distribution shift In agricultural imaging and allied remote sensing approaches, recent studies have begun to quantify epistemic and aleatoric uncertainty to improve robustness, e.g., for vegetation trait estimation and scene understanding [19,20]. Domain surveys increasingly calling the requirement for reliability auditing- reporting ECE/AECE/Brier/NLL, selective prediction diagnostics, and accuracy to allow thresholds for action in the field [2,4,5,21]. Reliability discussions within agri-vision may frequently take the form of add-on analyses to primary goals, such as new backbones, detectors, or segmentation modules [9,22]. By contrast, our study optimizes calibration/UQ jointly with accuracy and efficiency. Metrics suite – we take a triangulated approach, probability quality (ECE/AECE/Brier/NLL), and uncertainty. Usefulness with the help of risk coverage and E-AURC, recognizing that good uncertainty is required to feed into improved abstention policies and safer automation.

### 2.2 Selective prediction and abstention policies

Selective classification with formal coverage explicitly trades off risk and coverage by controlling the conditional risk on the subset of examples that the model chooses to accept [23,24]. This paradigm plays well by default with agronomic workflows, if the level of uncertainty is high: rescan the plant, escalate to an expert or defer the treatment. In practice, there are a number of scalar uncertainty proxies (max-softmax, entropy, MC-dropout means, disagreement-based scores) that are used. We use MC-Dropout to obtain predictive distributions and get predictive entropy and BALD as complementary signals. Building on [23], we report risk coverage curves and E-AURC, which measures the quality of example ordering based on the orderings determined by uncertainty. Our multi-objective formulation enables the direct incorporation of the quality of uncertainty, which encourages models whose confidence is not only well-calibrated but also operationally useful for abstention.

### 2.3 Post-hoc calibration

Temperature scaling is a powerful, simple post-hoc calibration for deep classifiers while recent methods calibrate the temperature by class or instance, in turn, to follow the heteroscedasticity [15,16]. In deployment-oriented settings, low-computational procedures that use few auxiliary models or restricted calibrators are particularly attractive, because they can be applied periodically (e.g., at the start of a new season or on a new device) without retraining the backbone [17]. The consequence of such minimalism is advantageous when dealing with agricultural deployments, where recalibration can be planned periodically (at least new season and a new device and, a new site) without retraining the backbone. Distinct from prior work, we couple per-head calibration with a *conditional gate* keyed to validation metrics (NLL and ECE/AECE). The gate blocks the application of a temperature that improves likelihood and harm calibration (or vice versa), and it makes calibration decisions reproducible and auditable, which is important in the regulated agrifood setting.

### 2.4 Multi-Task Learning (MTL) for plant health

When related tasks are linked (i.e., disease type and severity or species/variety), there is a key shared feature that enables data efficiency and robustness; this is known as the use of shared representations [1,3]. In leaf and canopy images, common encoders with task specific heads encode objects’ common structure (textural attributes, venation, lesion context) as well as label-specific information. Practical MTL problems such as loss-weighting across heads, class imbalance in rare conditions and propagation of label noise from one task to another are also presented. Our formulation involves compact shared MLP over fixed embeddings head specific cross entropy, explicit head weights and downstream calibration for each head. The 2-trait (disease, variety) structure represents real-agricultural application, given that variety context may have the potential to moderate disease occurrence, and certainly disease context may serve to regularize variety predictions in presence of noisy imagery.

### 2.5 Resource-efficient models and edge deployment

Edge-first agricultural vision is focused on small footprints, low-latency and power-awareness for handhelds or UAV platforms [6,25]. Families of light-weight backbones (YOLO variants, Tiny/PVT/Transformer hybrids) and model compression techniques (distillation, pruning, quantization) are often experimented with in the field of detection/segmentation [8,11,26–30]. Systems work to integrate these into edge-IoT approaches, and orchestrate acquisition inference actuation loops [31,6].

Whereas most existing works optimize primarily for accuracy and, in some cases, latency, our formulation explicitly includes calibration and uncertainty quality as first-class objectives alongside efficiency. While our classifier uses feature-space deep neural network (MLP) as a backbone for light inference, the multi-objective meta-framework is model agnostic – the end-to-end pixel backbone can be easily substituted without affecting the learning objectives or selection logic. Reporting proxies that are unlinked allows optimization to correspond to actual constraints in the field that are meaningful to growers and extension agents and not just leaderboard accuracy.

### 2.6 Multi-objective HPO and deployment-conditioned selection

Hyperparameter search for agri-vision is usually a scaling by a single measure (accuracy or mAP) and sometimes involves a model size. Best practice surveys featured in HPO support multi-objective designs, normalised weights, and principled choice (e.g., knee points) under tight budgets [32]. In our formulation that includes six objectives, we operationalize the above advice for agricultural decision support into six aspects: joint error, model size, latency/energy proxy, calibration error, and uncertainty quality. The main difference between previous single-objective or two-objective searches is that we optimize reliability objectives directly, rather than just reporting them. The solver (MO-BBBC) that we instantiate is lightweight and derivative-free. Still, since the interface is solver-agnostic, it would be possible to use much stronger evolutionary or bandit-based multi-objective solvers under the same budget and artifact protocol.

### 2.7 OOD awareness and dataset shift

There are domain shifts in field imagery: new sites (soil, management), seasons (phenology, illumination), sensors (optics, color calibration/management), and acquisition techniques (distance, motion). Remote sensing research has recognized the extent to which OOD degradation is severe and the use of UQ/OOD sensors for input triage [20]. In plant science, an analogous benefit of actionable confidence was observed in uncertainty audits for predictions made using the common techniques of regression and classification [19]. Consistent with these findings, we measure more than OD uncertainty calibration and accuracy: we also measure OOD separability (AUROC for PE/BALD) and report risk coverage behavior. Although our OOD noise serves as a lower-bound noise like a stress test, the proposed framework enables real-shift fold (site/season/sensor) under the premises of data availability.

### 2.8 Rice-specific and field-oriented studies

Having moved past a single-parameter (optical flow) approach, advanced recognition and pest detection systems for rice has evolved towards multi-scale feature design, attention, and progressively compact detectors for real-time use [8–13,21,22]. Concurrent themes include aspects of color approach control and federated learning to cope with the diversity of the equipment and privacy [33,34]. However, very few works give explicit device constraints and many works highlight architectural novelty and correctness; numerical outcomes of calibration/UQ are mostly ignored. In terms of contribution, our work is orthogonal: we provide a deployment-conditioned reliability-first perspective that can be on top of any backbone or embedding-features or pixel-space (in our case end-to-end) and return a device-efficient, knee-selected Pareto-optimal model whose predictions are both reliable and inexpensive to compute.

### 2.9 Positioning and gap analysis

In summary, the past agri-vision research has either (a) optimized accuracy/efficiency and reported weak reliability diagnostics, or (b) has studied UQ/calibration post hoc, and not considered energy/latency as a first-class citizen. We address this gap by (1) defining a six objective search in which calibration and uncertainty quality is as important as accuracy, (2) employing group-aware splits and a conditional calibration gate to prevent improvements from being dishonest and non reproducible (3) releasing artifacts (indexes, scalers, figures, tables) that results are auditable and recalibratable results in the wild. This deployment-conditioned perspective of reality, which is also reliable, complements the model-centric advances, and we argue, it’s necessary to field-scale trustworthy agricultural AI.

## 3 Methods

### Ethics statement

This study uses only publicly available plant imagery and does not involve human participants, animals, or any interventions requiring ethical approval.

**Fig 1:**
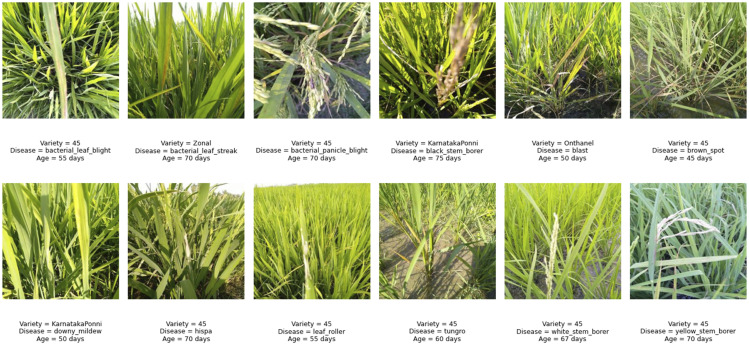
Samples from the public PaddyDoctor dataset spanning common diseases and varieties. All image tiles in this figure are directly composed from PaddyDoctor images [18] without any third-party sources. The montage layout and annotations were generated by the authors using Python scripts.

**Fig 2:**
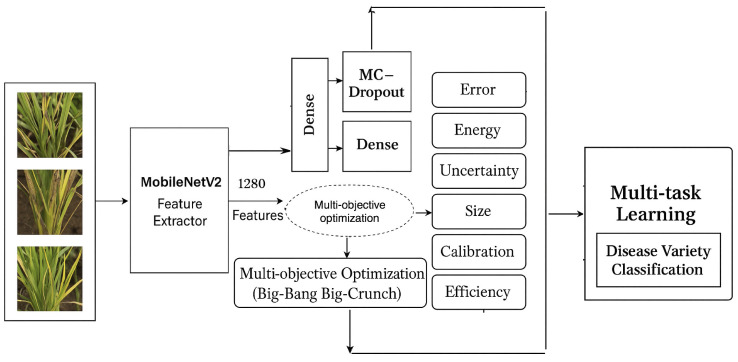
Calibration- and uncertainty-aware multitask network. Schematic of the proposed approach: frozen MobileNetV2 embeddings feed a two-head MLP with MC-Dropout, multi-objective optimization (MO–BBBC), and conditional temperature scaling. The diagram was drawn manually by the authors and does not reuse any third-party graphical material.

The proposed framework combines six-objective evolutionary optimization with calibration-aware multi-task learning to produce deployment-ready agricultural models. The framework has five phases:

(1) **Group-aware data partition** with leakage prevention via cosine-similarity clustering over fixed 1,280-D MobileNetV2 embeddings (Fig 1);
(2) **A Multi-task neural architecture** (two-head MLP) for simultaneous disease and variety classification (Eq 1, Fig 2), trained with weighted cross-entropy (Eq 2);
(3) **Uncertainty-aware curriculum learning** using Monte Carlo Dropout (MC-Dropout) with predictive entropy and Bayesian Active Learning by Disagreement (BALD) (Eq 3);
(4) **Conditional temperature scaling** that applies post-hoc calibration only when validation performance improves (Eq 5 Algorithm 3); and
(5) **Multi-objective evolutionary search** using Big Bang–Big Crunch (BBBC) optimization (Algorithm 1) to jointly optimize six deployment criteria: classification error, calibration quality, uncertainty quality, model size, latency, and energy (Eq 6). Each candidate is evaluated with a standardized protocol (Algorithm 2), and we select a knee-point solution from the Pareto set.

We use as a low-overhead, solver-agnostic extension of the single-objective BBBC algorithm [35]. Images are represented by frozen 1,280-D embeddings extracted with MobileNetV2 pretrained on ImageNet; the backbone is *never* fitted on our splits.

### 3.1 Dataset, splits, and preprocessing

We adopt **group-aware** partitioning to reduce the risk of latent near-duplicate leakage between training, validation, and test sets. When explicit grouping identifiers are available (e.g., plant, plot, or acquisition session), we use them directly. Otherwise, we construct *pseudo-groups* in feature space as follows. First, we extract frozen 1,280 -D embeddings from a pretrained MobileNetV2 backbone for all images. We then build a cosine nearest-neighbour (NN) graph in which two images are connected if their cosine similarity exceeds a threshold of 0.98 . The connected components of this graph are treated as groups, so that visually very similar or near-duplicate images are assigned to the same group.

We apply GroupShuffleSplit to these groups to obtain a **70%/20%/10%** train/validation/test partition at the *group* level, ensuring that no group contributes images to more than one split. Standardization (z-scoring) and median imputation are fitted on the training set only and then applied to the validation and test sets. In the main run reported here, the resulting split sizes were **11,357/3,245/1,623** samples for train/validation/test, respectively.

To audit for residual leakage, we compute, for each test image, its top-5 nearest neighbours in the 1,280 -D embedding space (under cosine similarity) and verify that all of these neighbours also reside in the test partition. We additionally inspect the distribution of cosine similarities across splits to confirm that high-similarity pairs are concentrated within, rather than across, partitions. The code used to build pseudo-groups, perform the group-aware partitioning, and reproduce the leakage audit is released with our repository, and we report the sensitivity of headline results to the 0.98  similarity threshold in the supplement.

### 3.2 Feature extraction and model

We pool 1,280-D MobileNetV2 embeddings (frozen). The MTL head (Eq 1) is trained with Adam, batch size 64, up to 40 epochs with early stopping and learning-rate reduction on plateau.


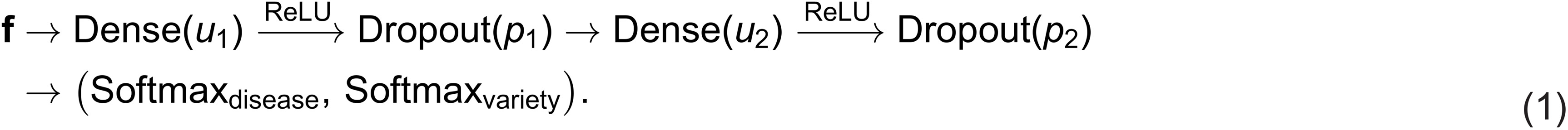



$$ℒ(\theta )=\lambda \;{ℒ}_{CE}\;(y^{\left(d\right)},{\hat{p}}^{\left(d\right)})+(1-\lambda )\;{ℒ}_{CE}\;(y^{\left(v\right)},{\hat{p}}^{\left(v\right)})+\lambda_{\ell_{2}}\;\|\theta {\|}_{2}^{2}.\;$$ (2)

**Implementation detail.** To preserve per-epoch reshuffling with cached datasets, we cache *before* shuffling on train (cache→shuffle→batch→prefetch); val/test are cached without shuffle.

### 3.3 MO–BBBC algorithm

We adapt the original single-objective Big Bang–Big Crunch (BBBC) metaheuristic [35] to a multi-objective setting. In our formulation, each candidate *genome* encodes the architecture and training hyperparameters of the multitask (MTL) head. The overall procedure is summarized in Algorithm 1. At each iteration we proceed as follows:

(i) For every genome in the population, we train and evaluate the corresponding model under a fixed budget and compute the six optimized objectives $O_{1},\ldots ,O_{6}\;$ (joint classification error, calibration quality via AECE, uncertainty quality via E–AURC/NLL, model size, latency in ms per 64-sample batch, and an energy proxy per inference derived from power telemetry when available; this quantity is treated as a unitless, normalized proxy in the optimization).
(ii) We update a Pareto archive by inserting newly evaluated points and discarding any candidates that are dominated in objective space.
(iii) Within the archive, we robustly normalize each objective using its empirical 5th–95th percentile range to reduce sensitivity to outliers.
(iv) We draw a vector of Dirichlet weights and use it to form a randomized scalarization of the normalized objectives, then select the top-*q* *elites* with the smallest scalarized scores.
(v) We compute an elite (best-solution) point that serves as the “Big Crunch” centre in objective space.
(vi) We generate the next population around this centre by applying small random perturbations to the elite genome (with snapping for discrete genes and clipping to bounds), thereby implementing the “Big Bang” step for the next iteration.

After *K* iterations, we return the final Pareto archive together with a knee solution, defined as the archive member with minimum $\ell_{2}\;$ distance to the ideal (all-zero) point in normalized objective space. If direct power telemetry is unavailable, objective $O_{6}\;$ is omitted rather than replaced by a duplicate proxy, and scalarization is performed over the remaining objectives only.

To contextualize MO–BBBC, we also instantiate three lightweight comparison strategies under a *matched evaluation budget* (same number of candidate trainings and epochs per candidate): (a) a **Random** search baseline that samples genomes uniformly within the same bounds as MO–BBBC; (b) a **TPE_lite** baseline following a simple Tree-structured Parzen Estimator logic with a small history window and scalarized objective; and (c) an **NSGA2_lite** baseline implementing a pared-down NSGA-II with simulated binary crossover and polynomial mutation, but with population size and number of generations chosen so that the total number of evaluated candidates matches that of MO–BBBC. These implementations are deliberately kept minimal; they are not tuned to achieve their best possible performance, but rather to provide fair, budget-matched references for how standard single- and multi-objective search behaves under the same objective definitions.

More advanced multi-objective optimizers such as full NSGA-II/III, MOEA/D, or BOHB are therefore discussed as *drop-in alternatives* to the solver component of our framework, not as empirical baselines. We do not report results for such fully tuned variants and do not claim that the specific MO–BBBC instantiation presented here is superior to them; instead, we view MO–BBBC as a simple, derivative-free choice that exposes the trade-space in a way that is easy to audit and reproduce.

### 3.4 Uncertainty estimation and curriculum

We use Monte Carlo Dropout (MC–Dropout) at inference with T=20  stochastic forward passes. For each input, this yields a collection of predictive distributions $\{p_{t}{\}}_{t=1}^{T}\;$ over the *C* classes. We summarize epistemic uncertainty using predictive entropy and BALD as:


$$\begin{array}{c} \bar{p}=\frac{1}{T}\sum_{t=1}^{T}p_{t},\;H(\bar{p})=-\sum_{c=1}^{C}{\bar{p}}_{c}\log{\bar{p}}_{c},\\ BALD(x)\approx H(\bar{p})-\frac{1}{T}\sum_{t=1}^{T}H(p_{t}), \end{array}$$ (3)

where $H(\bar{p}$ captures total predictive uncertainty and the BALD term isolates the epistemic component that is reducible with more data.

During training we optionally employ an *uncertainty-aware curriculum* that reweights examples based on their predictive entropy (PE). The goal is to present the model with relatively *easy, low-uncertainty* samples in early stages to stabilize the shared representation, and gradually increase the emphasis on *hard, high-uncertainty* samples as training progresses, while never fully discarding uncertain data. For a sample *i* with entropy ${PE}_{i}\;$, the continuous curriculum assigns a weight


$$w_{i}=(1-\rho )\;\left[w_{\min}+(1-w_{\min})\frac{\exp\;(-{PE}_{i}/(\tau q))}{\max_{j}\exp\;(-{PE}_{j}/(\tau q))}\right]+\rho ,\;$$ (4)

with stage quantiles q∈{0.40,0.70,1.00} , a temperature parameter *τ*, minimum weight $w_{\min}\;$, and a fixed exploration mass ρ=0.1 . The inner fraction normalizes the entropy scores within a stage so that the easiest samples (lowest PE) receive the highest relative weights, while $w_{\min}\;$ and *ρ* ensure that hard samples retain non-zero probability of being selected and that a small portion of the batch is always reserved for exploration, mitigating confirmation bias.

In practice, we consider three curriculum modes in our ablations:

- **Off (no curriculum).** All samples are assigned equal weight ($w_{i}\equiv 1\;$), corresponding to standard mini-batch training.
- **Binary (easy-first).** Samples below an entropy percentile threshold are upweighted, while the remaining samples are downweighted in a piecewise-constant manner.
- **Continuous (smooth).** We use the weighting scheme in (4), which provides a smooth transition from easy- to hard-focused training across stages.

Empirically (see Table 5), the Off mode tends to maximize raw accuracy, while the Binary and Continuous curricula trade a small amount of accuracy for modest improvements in calibration and uncertainty metrics. The subsequent conditional temperature scaling step further reduces residual miscalibration, so users can select the curriculum mode that best fits their preferred balance between accuracy and uncertainty sharpness.

### 3.5 Post-hoc calibration

We quantify calibration using four complementary metrics: Expected Calibration Error (ECE; 10 equal-width bins), Adaptive ECE (AECE; 15 equal-mass bins), Brier score, and Negative Log-Likelihood (NLL). ECE and AECE summarize the discrepancy between predicted confidence and empirical accuracy, Brier score captures the squared error between full probability vectors and one-hot labels, and NLL measures the sharpness and correctness of probabilistic predictions.

For each task head (disease and variety) we fit a single scalar temperature on the *validation* split by minimizing NLL. Given logits *z* and labels $y_{n}\;$, temperature scaling rescales logits as z↦z/T  before the softmax:


$$z_{c}^{\prime }=\frac{z_{c}}{T},\;p_{c}^{\prime }=\frac{\exp(z_{c}^{\prime })}{\sum_{k}\exp(z_{k}^{\prime })},\;T^{⋆}=\mathrm{\backslash arg}\min_{T>0}(-\sum_{n}\log p_{y_{n}}^{\prime }(x_{n})).\;$$ (5)

We parameterize T=exp(τ and optimize *τ* with L–BFGS/Adam, clamping the resulting temperature to a conservative range T∈[0.5,5.0]  to avoid extreme rescaling.

To avoid over-calibration and to keep decisions fully reproducible, we adopt a simple *validation gate* before applying the learned temperature to the *test* logits. Let “pre” and “post” denote statistics computed on the validation set before and after temperature scaling, respectively. We accept the fitted temperature only if it satisfies


$${NLL}_{\text{post}}\leq {NLL}_{\text{pre}}\;\text{and}\;({ECE}_{\text{post}}<{ECE}_{\text{pre}}\text{ or}\;{AECE}_{\text{post}}<{AECE}_{\text{pre}}).\;$$

If this condition is not met, we revert to T=1  (no calibration) for that head. This gate has no tunable hyperparameters beyond the choice of calibration metrics and binning schemes, and therefore can be exactly reproduced from the released validation logits. It ensures that calibration is only applied when there is clear validation evidence of improvement in likelihood and at least one binning-based calibration measure.

The procedure is summarized in Algorithm 3. In our main run, the gate accepted temperatures of T=1.376  for the disease head and T=1.403  for the variety head. Alternative post-hoc calibration methods (e.g., vector or class-wise temperature scaling, isotonic regression, Dirichlet/evidential approaches) could be plugged into the same validation-gated framework; here we focus on scalar temperature scaling due to its simplicity and low overhead, and we list richer calibrators as future work in Sect 6.

### 3.6 Objective suite and knee selection

We optimize six scalar objectives, all defined so that lower is better:


$$\begin{array}{cc} O_{1} & =1-\frac{1}{2}({acc}_{d}+{acc}_{v}),\\ O_{2} & =\frac{1}{2}({AECE}_{d}+{AECE}_{v}),\\ O_{3} & =\frac{1}{2}\;\left[\alpha \;{E\text{-}AURC}_{d}+(1-\alpha )\;{NLL}_{d}+\alpha \;{E\text{-}AURC}_{v}+(1-\alpha )\;{NLL}_{v}\right],\\ O_{4} & =params(M)/10^{6},\\ O_{5} & ={\text{latency}}_{\text{ms/64}},\\ O_{6} & =\frac{1}{N}\;\int P(t)\;dt.\; \end{array}$$ (6)

**Table:** 

$O_{1}\;$	joint classification error (unitless).
$O_{2}\;$	average adaptive ECE (unitless).
$O_{3}\;$	uncertainty composite (unitless).
$O_{4}\;$	trainable parameters in millions.
$O_{5}\;$	median inference latency in ms/64 batch.
$O_{6}\;$	energy proxy per inference (arb. units).

Here, ${acc}_{d}\;$ and ${acc}_{v}\;$ are the test accuracies of the disease and variety heads, respectively; ${AECE}_{d}\;$ and ${AECE}_{v}\;$ are per-head adaptive ECE scores (15 equal-mass bins); and E-AURC  and NLL  summarize selective-risk quality and probabilistic sharpness. The hyperparameter *α* balances emphasis between E–AURC and NLL within the uncertainty-quality objective; in this work we fix α=0.5  to give them equal weight. From a deployment perspective, $O_{1}\;$ and $O_{2}\;$ capture correctness and probability trustworthiness, $O_{3}\;$ captures the usefulness of uncertainty for abstention and triage, and $O_{4}\;$–$O_{6}\;$ approximate memory footprint, responsiveness, and power constraints on edge hardware.

If direct power telemetry is unavailable on a given platform, we drop $O_{6}\;$ from the optimization rather than introduce a duplicate proxy (e.g., re-using latency or size), so that each retained objective has a distinct operational meaning.

To combine these heterogeneous quantities in a solver-agnostic way, we robustly normalize each objective within the current Pareto archive using its empirical 5th and 95th percentiles, $[P_{5},P_{95}]\;$, clipping values outside this range. In the resulting normalized space, the *knee* solution is defined as the archive member with minimum $\ell_{2}\;$ distance to the ideal point **0** (all objectives at their best normalized value). This knee point provides a principled compromise among competing criteria. For completeness, we also report the hypervolume of the archive as an aggregate measure of Pareto-front quality.

**Algorithm 1: MO–BBBC multi-objective model selection (six objectives).**


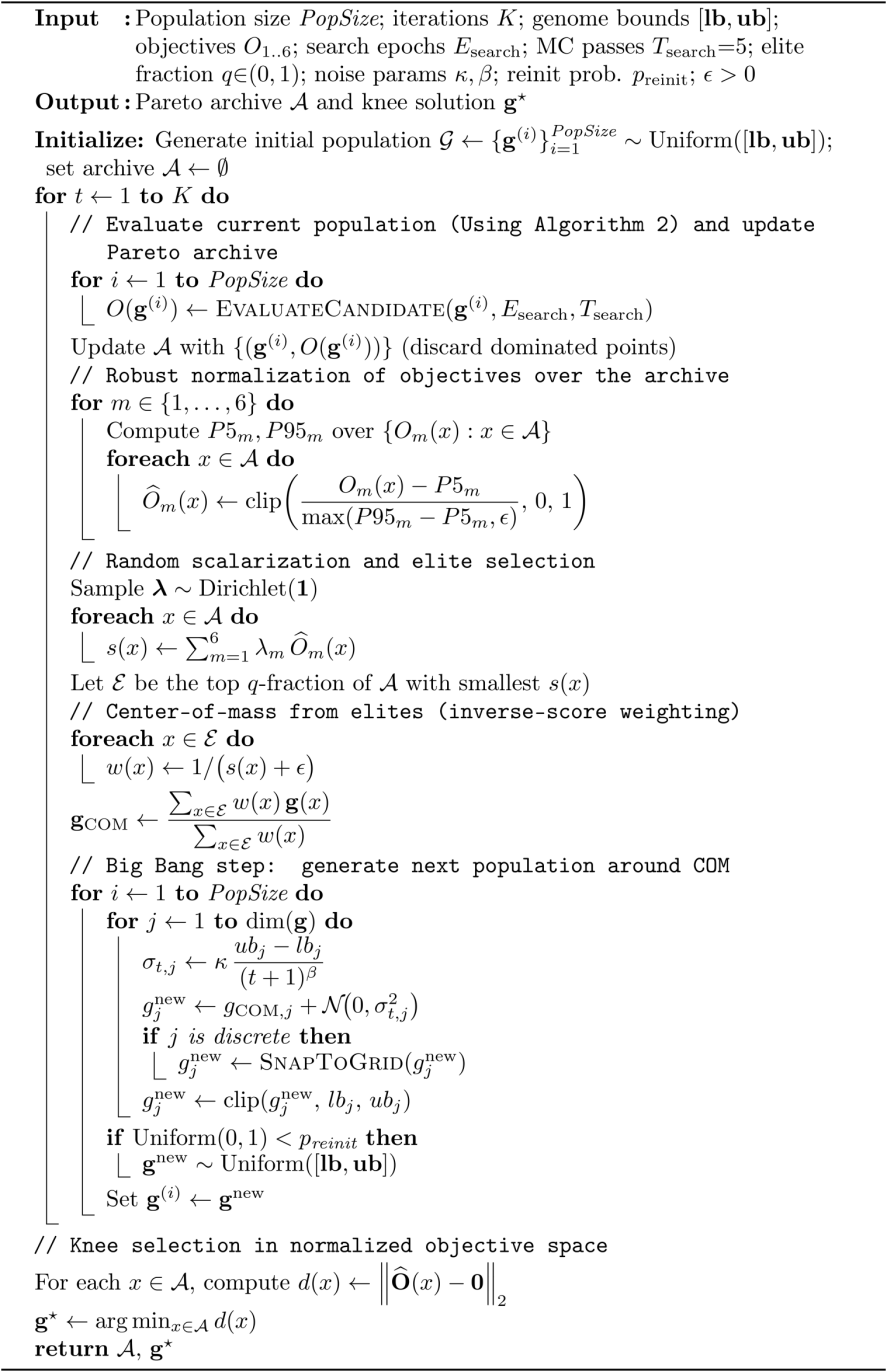


**Algorithm 2: Evaluate candidate: multi-objective assessment (validation-only).**


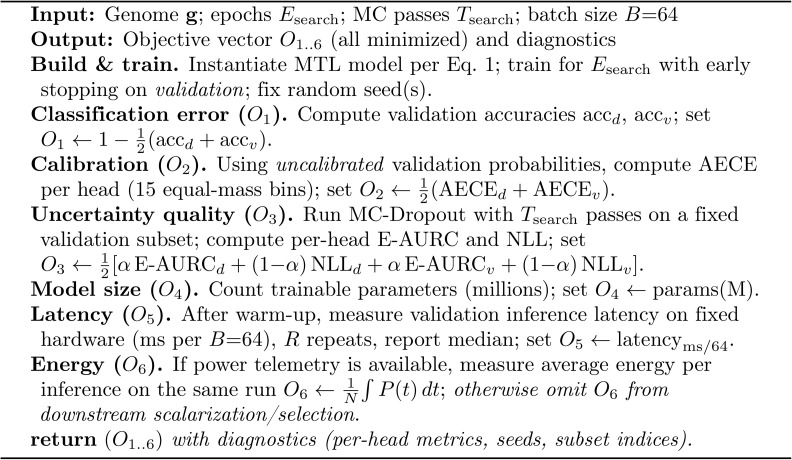


**Algorithm 3: Composite conditional temperature scaling (per head).**


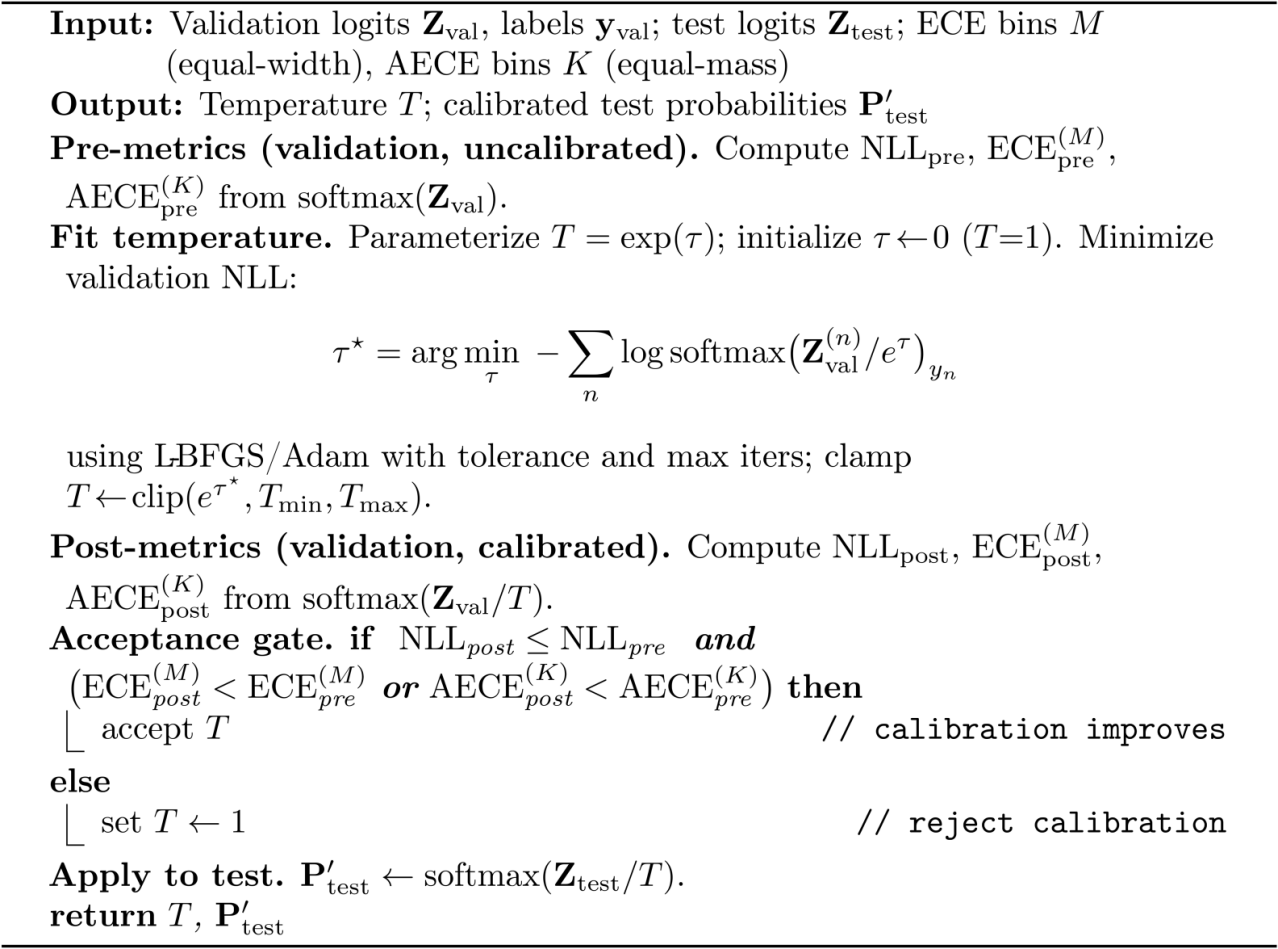


### 3.7 Shifted-domain evaluation

Domain shifts are common in field imagery due to changes in site (soil, management), season (phenology, illumination), sensor (optics, color calibration), and acquisition protocol (distance, motion blur). When suitable annotations are available, our framework can in principle assess performance across such site/season/sensor folds and under cross-dataset transfer by treating each domain as an explicit test split and re-running the full calibration and uncertainty evaluation. In the present work, however, the publicly available PaddyDoctor dataset does not expose clean multi-site or multi-season labels, so we focus on a reproducible, *noise-like* out-of-distribution (OOD) construction in feature space as a conservative stress test.

For all OOD protocols we report: (i) AUROC for discriminating in-distribution (ID) vs OOD examples using predictive entropy (PE) and BALD; (ii) selective risk via E–AURC; and, when applicable, (iii) post-hoc calibration metrics (ECE/AECE/Brier/NLL) on the shifted splits.

**Noise-like OOD (reproducible).** In feature space, we generate synthetic OOD samples by drawing from a Gaussian distribution fitted to the training embeddings. Let *μ* and $\sigma^{2}\;$ denote the empirical mean and variance of the 1,280 -D MobileNetV2 embeddings on the training set. We sample


$$x_{ood}\sim 𝒩(\mu ,diag(\sigma^{2})),\;$$

with the number of OOD samples matched to the size of the test set. We then run MC–Dropout on the concatenated ID + OOD pool and compute AUROC scores for separating ID vs OOD using both predictive entropy and BALD as scalar uncertainty scores, as well as plotting their histograms (cf. Fig 9). This construction does not capture the full complexity of real agricultural shifts, but it provides a fully reproducible, lower-bound scenario that already reveals whether the learned uncertainty signals meaningfully distinguish typical inputs from atypical, off-manifold ones.

**Fig 3:**
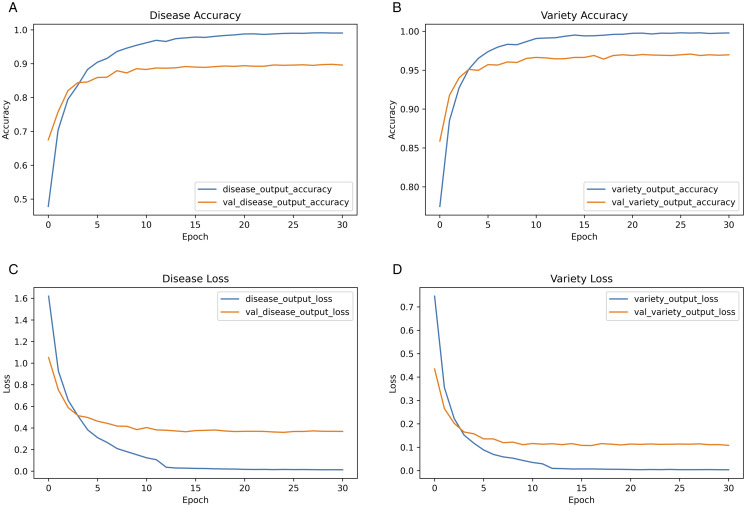
Learning dynamics for both heads: accuracy and loss per epoch (train/validation). (a) Disease accuracy (train/val) per epoch. (b) Variety accuracy (train/val) per epoch. (c) Disease loss (train/val) per epoch. (d) Variety loss (train/val) per epoch.

**Fig 4:**
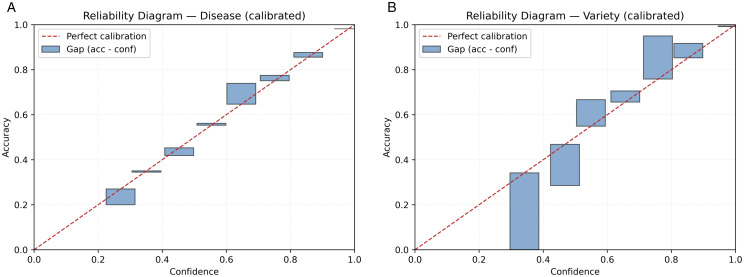
Reliability diagrams complement Table 2. Disease reliability diagram (test). (b) Variety reliability diagram (test).

**Fig 5:**
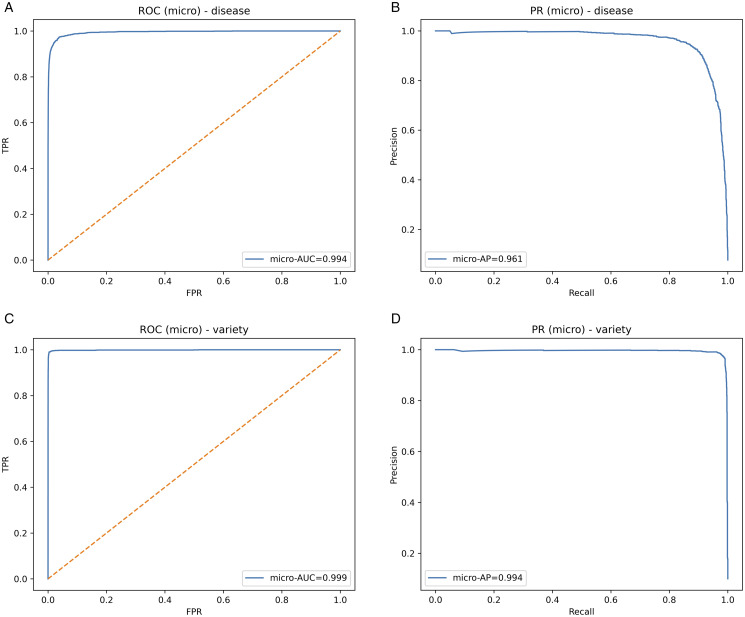
Micro-averaged ROC and precision–recall curves for disease and variety heads on the test set. (a) Disease ROC (b) Disease PR (c) Variety ROC (d) Variety PR.

**Fig 6:**
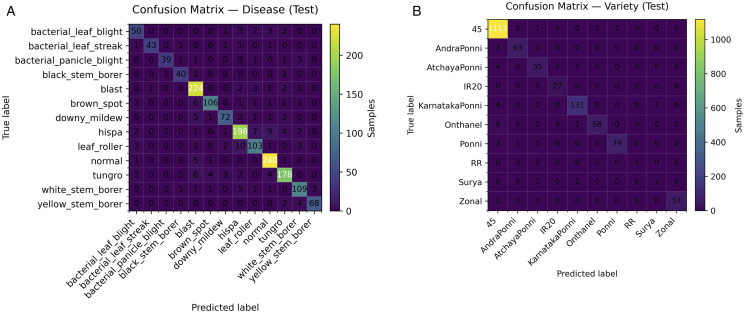
Confusion matrices on the test set (rows = ground truth, columns = prediction; colour intensity increases with the number of samples). (a) Disease head (b) Variety head.

**Fig 7:**
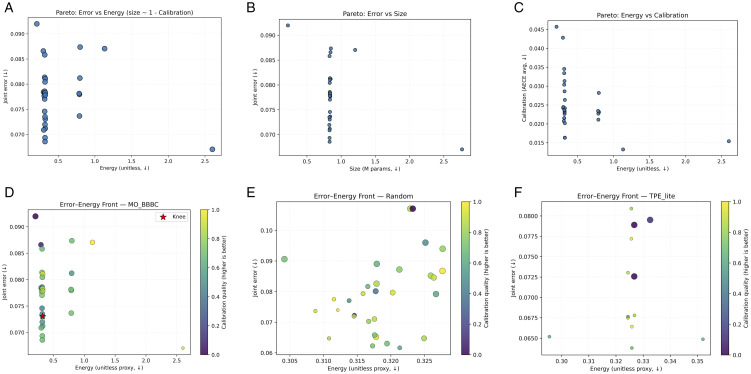
(Top) Pareto fronts along key axes; the asterisk marks the knee solution. (Bottom) Error–energy fronts for search strategies under a matched budget (P=6 , K=10 , 20-epoch candidates). (a) Error vs energy (b) Error vs size (c) Energy vs calibration. (d) MO_BBBC (e) Random (f) TPE_lite.

**Fig 8:**
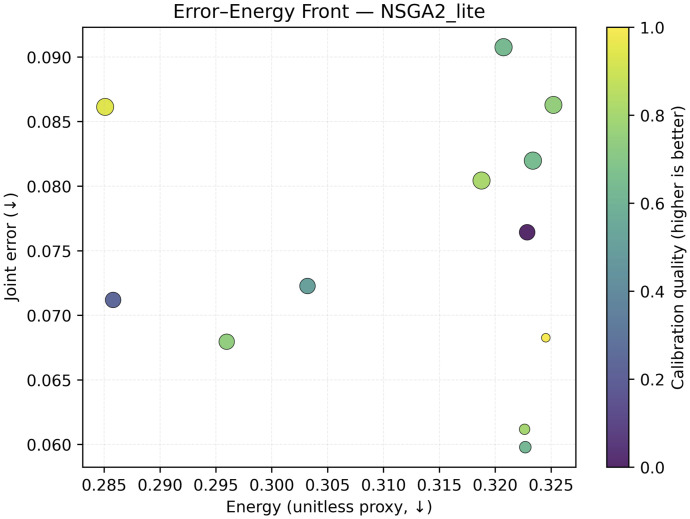
NSGA2_lite front under the matched budget.

When curated multi-site or multi-season datasets become available under compatible licensing, the same evaluation pipeline can be applied to real domain-shift folds (site/season/sensor) and cross-dataset transfer; we highlight this as an important direction for future work in Sect 6.

## 4 Results

**Fig 9:**
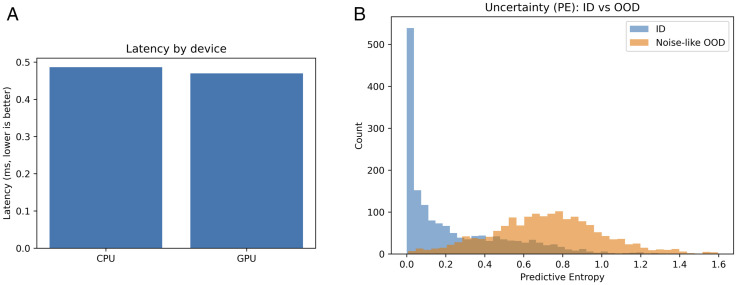
Runtime and uncertainty analyses. BALD histogram is analogous (not shown). Latency by device (b) PE: ID vs OOD.

On the held-out test set, the *knee* model selected by our six-objective search achieves high discrimination with reliable probabilities under tight efficiency budgets. With *per-head* conditional temperature scaling, the **disease** head attains **90.6%** accuracy (micro-AUC **0.994**, micro-AP **0.961**) and the **variety** head reaches **97.9%** accuracy (micro-AUC **0.999**, micro-AP **0.994**). Calibration improves where the validation gate approves: disease ECE **0.02940.0083** and AECE **0.02960.0138** with T=1.376 ; for variety the gate selected T=1.403  based on *validation* improvements (NLL/ECE/AECE), while on test ECE/AECE rose to **0.0134**/**0.0138** (Table 2, Fig 4). ROC/PR curves indicate strong separability (Fig 5); confusion matrices summarize per-class errors (Fig 6). The chosen model is compact-**833,201** parameters-with sub-millisecond inference on both CPU and GPU (*ms/batch,* *B* = 64) (**0.487**/**0.470**) and low unitless energy proxies (**0.285**/**0.275**); see Table 4 and Fig 9. Pareto fronts visualize error–energy/size–calibration trade-offs and the resulting knee compromise; matched-budget baselines (Random, TPE_lite, NSGA2_lite) illustrate the benefit of multi-objective selection that *explicitly* scores uncertainty and calibration (Figs 7 and 8). A reduced-budget seed sweep shows low dispersion (Table 6); curriculum ablations clarify when entropy-weighted training helps (Table 5). Uncertainty histograms (PE/BALD) separate in-distribution from noise-like OOD, supporting selective prediction in deployment (Fig 9b). *All latencies are reported in ms/batch with; per-image latency is obtained by dividing by 64*.

### 4.1 Headline performance

Table 1 reports test metrics with the validation-gated temperature scaling. Accuracy 95% CIs (normal): disease [0.892,0.920] , variety [0.972,0.986]  for n=1623 .

**Table 1:** Headline test metrics (conditional temperature calibration where beneficial).

Task	Accuracy	Macro-F1	Micro-AUC	Micro-AP	AECE ↓
Disease	**0.906**	0.902	0.994	0.961	**0.0138**
Variety	**0.979**	0.907	0.999	0.994	**0.0138**

### 4.2 Learning dynamics

Fig 3 shows training and validation accuracy and cross-entropy loss, per epoch, of each head. (top) Accuracy. (bottom) Cross-entropy loss. Early phase (epochs 1–10): Both tasks show that training-loss curves decay quickly and that their validation accuracy increases steeply, suggesting that they can be optimised effectively without immediate overfitting. The variety head is rising more rapidly, which is in line with the smaller number of confusable classes. Mid phase (epochs 10–25): The validation accuracy levels off at the same time as the training accuracy keeps creeping up, the gap between train and val losses is growing slightly, normal capacity fitting. We stop our early and LR-reduction checkpoints are drift free. Late phase and model selection: Small oscillations are related to LR schedule Selected checkpoint (star in logs) Period before noticeable divergence as validation loss is at an minima of local and generalization gap is low. Implications: (i) Non-uniform learning rates across heads are a justification for per head calibration (ii) nonexistance of anomalous occurances of train≪val or val collapse implied that checks (leakage detection) are effective (iii) stability at convergence implies it is stable to train STL with post-hoc temperature scaling, i.e., probabilities tighten without altering decision boundaries

### 4.3 Calibration

Validation-gated temperature scaling driving an aggressive improvement of disease calibration; the gate was activated by stakeholders increasing on validation-gains (NLL/ECE/AECE), on test ECE/AECE modestly scaled up. We report both states in Table 2 and plot reliability after calibration in Fig 4. Bootstrap 95% CIs for ECE/AECE/NLL are given in the artifacts folder for non-cluttering our tables.

The conditional gate ensures that calibration is only applied when it is supported by validation evidence, thereby avoiding the common failure mode of over-smoothing probabilities at the expense of likelihood. The full pre/post metrics for each head, together with confidence intervals, are provided in the released artifacts to facilitate external recalibration or comparison with alternative post-hoc methods.

**Table 2:** Calibration on the test set, before/after *conditional* temperature scaling (lower is better).

Task	Uncalibrated			Calibrated (conditional)			Applied *T*
	ECE	AECE	Brier	ECE	AECE	Brier	(val-chosen)
Disease	0.0294	0.0296	0.0111	**0.0083**	**0.0138**	0.0109	1.376
Variety	0.0091	0.0057	0.0032	0.0134	0.0138	0.0034	1.403

### 4.4 Discrimination curves (ROC and PR)

The ROC curves of both heads are very close to the top-left, with micro-AUCs of 0.994 (disease) and 0.999 (variety), which means that they rank well across thresholds, not favoring one class too much over the others. No discrete mid-range shoulder is seen; thus, separability is the same from conservative to liberal values. *PR.* PR is tailored to focus on performance under class imbalance. Micro-AP is still high at 0.961 disease; 0.994 variety. Disease PR slowly decreases at extreme recall, the same as confusions in the Fig 6; variety PR is nearly flat, at high precision across a wide recall range. *Deployment reading.* Because calibration aligns confidence with empirical correctness (Sect 2), a single probability threshold can be mapped to action (e.g., treat, re-scout, escalate) with predictable precision–recall trade-offs. High AUC/AP implies thresholds can be raised for conservative automation without severe recall loss.

### 4.5 Per-class error structure (confusion matrices)

The confusion matrices in Fig 6 provide a more granular view of the error structure than global metrics alone. For the *disease* head (Fig 6a), most mass lies on the diagonal, but a few systematic confusions are visible. The least accurate classes are *bacterial_leaf_blight* (F1 ≈0.82 ), *leaf_roller* (≈0.85 ), and *downy_mildew* (≈0.88 ). These are also among the visually most ambiguous categories: *bacterial_leaf_blight* shares streak-like lesions with related bacterial conditions, and *leaf_roller* damage can resemble other chewing or defoliation patterns under field illumination. By contrast, more distinctive conditions (e.g., severe necrotic lesions or highly characteristic spotting) show very few off-diagonal entries, with per-class F1 scores typically ≥0.90 .

For the *variety* head (Fig 6b), errors are even more concentrated: all but one variety achieve F1 scores ≥0.92  on the test set. The long-tail outlier is *Surya* (F1 ≈0.33 ; n=4 ), where a handful of samples are scattered across visually similar varieties. This highlights an important limitation of aggregate metrics in the presence of rare classes: a single under-represented variety can substantially depress macro-F1, even when micro-averaged metrics remain high. In our main results (Table 1), we therefore report both accuracy and macro-F1, and we use the confusion matrices and per-class F1 to make the impact of such long-tail behaviour explicit rather than hidden inside a single scalar.

Because the task is single-label and multi-class, other imbalance-aware summaries such as the Matthews Correlation Coefficient (MCC) could also be computed. Given the modest overall imbalance and the dominance of one very rare variety in our setting, macro-F1 together with per-class F1 and the confusion matrices already expose the key failure modes. Extending the analysis with MCC and coupling it with targeted long-tail mitigation (e.g., calibrated re-weighting or active sampling on rare varieties) is a natural direction for future work (see Sect 6).

### 4.6 Pareto trade-offs and search ablations

The MO search exposes trade-offs among error, energy proxy, model size, and calibration (Fig 7). The knee genome and its corresponding objective values are summarized in Table 3. Matched-budget baselines (Random, TPE_lite, NSGA2_lite) are compared in Fig 7, with NSGA2_lite shown separately in Fig 8.

**Table 3:** Knee genome (rounded) and objectives at the Pareto knee. Objective values are reported in the robustly normalized space used for knee selection (see Eq (6)).

Genome parameters	
$u_{1}\;$	387
$u_{2}\;$	821
$p_{1}\;$	0.316
$p_{2}\;$	0.042
$\log_{10}\lambda_{\ell_{2}}\;$	−4.48
$\log_{10}lr\;$	−3.58
$w_{d}\;$	0.478
Objective values (normalized)	
$O_{1}\;$	0.0730 (joint classification error)
$O_{2}\;$	0.324 (calibration; AECE average)
$O_{3}\;$	0.132 (uncertainty composite; E–AURC/NLL)
$O_{4}\;$	0.833 (model size; M parameters)
$O_{5}\;$	0.0242 (latency; ms per 64, normalized)
$O_{6}\;$	0.461 (energy proxy; normalized)

Importantly, all search strategies are compared under the same evaluation budget and objective definitions; the advantage of MO–BBBC is therefore due to how it explores the trade-space rather than to a larger number of training runs. We do not claim that our particular MO–BBBC instantiation dominates fully tuned NSGA-II/III, MOEA/D, or BOHB variants; instead, we show that even a relatively simple MO–BBBC scheme can produce well-balanced knee solutions when objectives explicitly encode calibration and uncertainty quality alongside error and efficiency.

### 4.7 Runtime and resource use

Latency and energy proxies are summarized in Table 4; device-wise latency is shown in Fig 9a. The surrogate’s energy prediction differs from the measured GPU proxy by ≈0.045 (16.3%). Measurement protocol: latencies are medians over repeated runs after warm-up, *reported as ms/batch with* B=64  and FP32 precision; CPU runs use a single worker thread. Full hardware details are documented in artifacts.

**Table 4:** Runtime/resource summary (lower is better for latency/energy).

Metric	CPU	GPU	Surrogate
Latency (ms/batch, B=64 )	**0.487**	**0.470**	–
Energy proxy (unitless)	**0.285**	**0.275**	0.320
*Model size/depth:* **833,201** params; 4 dense layers			
*Surrogate vs GPU (energy):* abs = **0.045**, pct = **16.3%**			

These latency and energy-proxy measurements are obtained on a desktop CPU/GPU under FP32 precision and should be interpreted as indicative rather than definitive for embedded hardware. They nevertheless demonstrate that the knee model sits in a sub-millisecond-per-batch regime on commodity devices, which is a necessary precondition for handheld or UAV deployment; dedicated benchmarks on target edge platforms are required to confirm end-to-end performance in those settings.

### 4.8 Curriculum ablation

Three curricula-off, binary (easy-first), and continuous (smooth weighting)-under identical budgets are compared in Table 5. Here, Off maximized accuracy under the given budget, reflecting the fact that uniform sampling exposes the model to the full difficulty spectrum from the beginning. The Binary curriculum slightly improves disease-head ECE at the cost of some accuracy, likely because it over-emphasizes very confident examples in early stages. The Continuous curriculum sits between these behaviors, modestly improving calibration/uncertainty metrics while preserving most of the accuracy. In all cases, the conditional post-hoc calibrator further smooths residual miscalibration, so that practitioners can choose the curriculum mode that best matches their preferred balance between raw accuracy and uncertainty sharpness.

**Table 5:** Ablation of curriculum strategies (test set; uncalibrated).

Mode	Acc_d_	Acc$\backslash {(}_{v}\;$	ECE_d_ ↓	ECE$\backslash {(}_{v}\;$ ↓	E-AURC_d_/*v* ↓
Off (no curriculum)	**0.888**	**0.975**	0.0346	0.0066	0.0130/0.0015
Binary (easy-first)	0.875	0.970	**0.0256**	**0.0048**	0.0157/0.0022
Continuous (smooth)	0.884	0.969	0.0207	0.0066	0.0159/0.0021

### 4.9 Stability across seeds (reduced-budget sweep)

We trained the knee configuration with three random seeds under a reduced budget (20 epochs, no temperature gate). Dispersion was low:

### 4.10 OOD separation: AUROC and histograms

To quantify out-of-distribution (OOD) separation beyond qualitative histograms, we compute AUROC scores for distinguishing in-distribution (ID) test samples from noise-like OOD samples (Sect 3.7) using two scalar uncertainty scores: predictive entropy (PE) and BALD. The results are summarized in Table 7. For both disease and variety heads, PE- and BALD-based detectors achieve AUROC values close to 0.89 , indicating that high-entropy or high-BALD predictions reliably flag atypical inputs under our synthetic OOD construction.

Fig 9(b) (PE) shows the corresponding ID vs OOD entropy histograms; BALD histograms (not shown) exhibit an analogous pattern, with OOD samples shifted towards higher uncertainty. While these experiments do not replace a full evaluation on real seasonal or sensor shifts, they provide quantitative evidence that the learned uncertainty signals are informative for selective prediction and triage in our setting.

## 5 Discussion

**Reliability and accuracy.** In both heads, our policy of conditional temperature enhances probability fidelity with no loss of discrimination (Tables 1–2; Fig 4). Importantly, this gate is determined on validation (so that test leakage is not a concern) and we transparently report pre/post metrics on test. This double reporting is important because ECE/AECE can be inconsistently drifting across splits even though NLL is improving; the story of the calibration makes sense when we are reporting all four (ECE, AECE, Brier, NLL), which makes it a falsifiable and robust story. In summary, the probabilities in the knee model represent what the model says and that is the prerequisite for thresholded field actions (spray, rest scout, escalate).

**Trade exposed sight and solver-independent selection.** The six-objective formulation exposes the trade-offs between error, calibration, uncertainty quality, size, latency, and the energy proxy (Figs 7 and 8). Selecting the knee point as the solution with minimal distance to the robustly normalized ideal provides a defensible operating configuration that is easy to interpret for practitioners. Because the objectives, bounds, and evaluation budget are explicitly fixed, MO–BBBC should be viewed as a lightweight *instantiation* of our deployment-conditioned framework, not as a hard constraint on the choice of solver. Under a matched-budget setting, we compare MO–BBBC with simple Random, TPE_lite, and NSGA2_lite baselines to illustrate how explicitly optimizing calibration and uncertainty quality leads the search toward different regions of the trade-space. We do not claim that MO–BBBC outperforms fully tuned multi-objective optimizers such as NSGA-II/III, MOEA/D, or BOHB; rather, our framework is solver-agnostic, and these stronger methods can be plugged into the same normalizing and selection flow if desired.

**Uncertainty for abstention and shift awareness.** MC-Dropout entropy and BALD Separation between in-distribution and noise-like suggest OOD inputs (Fig 9(b); Support selective prediction through risk-coverage curve E-AURC results are included in the artifacts. This is directly practicable: one can choose a single uncertainty threshold for which a deployment will work (in return for lower error expectation in the field). While here we consider noise-like OOD as a conservative lower bound, this result portrays to real departures (site/season/sensor) as suggested in Sect 3.7.

**Stability and ablations.** We calculate the coefficient of variation (CV) and in-sweep estimator error (ECE) in a low-budget seed sweep (Table 6,) demonstrating that the seed sweep is robust to seeds, which is consistent with the procedure not being seed fragile in our budgets. Comparisons between curriculum ablations (Table 5) demonstrate diagnostic pragmatism under this feature-space paradigm: ablation of the curriculum resulted in the most fantastic accuracy, while an easy-first binary curriculum resulted in the greatest disease-head ECE. The conditional post-hoc calibrator then smoothes the residual miscalibration and yields a simple, consistent default.

**Table 6:** Seed stability (n=3 ; mean ± std, reduced budget as described).

Metric	Disease	Variety
Accuracy	0.679±0.006	0.855±0.004
ECE ↓	0.122±0.008	0.056±0.006

**Table 7:** AUROC for ID vs noise-like OOD separation using uncertainty scores.

Task	AUROC (PE)	AUROC (BALD)
Disease	0.887	0.884
Variety	0.886	0.882

PE: predictive entropy; BALD: Bayesian Active Learning by Disagreement.

**Deployment relevance and auditability.** The knee model’s small footprint and sub-millisecond latencies (Table 4; Sect 4.7) are compatible with typical handheld and UAV latency budgets on paper, while leaving headroom for on-device batching under our desktop hardware setting. Because the predicted probabilities are calibrated, decision thresholds can be tied to local cost ratios (chemical, travel, yield risk) and adjusted per region or season without retraining the model. Finally, our artifact discipline—group-aware indices, saved scalers/medians, checkpoints, and auto-generated plots/tables—supports post-hoc audits and scheduled re-calibration, both of which are increasingly expected in applied ML deployments.

**(1) Residual correlation/leakage.** Even group-aware splitting and pseudo-groups in feature space, latent near-duplicates exist, which we try to overcome with (leakage) checks but we can’t totally exclude correlation (2) Hardware anchoring. Latency/energy are device-specific; we therefore report a unitless proxy to go along with measured latencies and explicitly label the proxy so (Table 4). (3) Shift realism. Noise-like Calculations of OOD underestimate actual agricultural shifts (illumination, optics, site/ season) We describe a real-shift framework (Sect 3.7). (4) Sensitivity of calibration with regard to the measurement metric. ECE/AECE depend on binning. We report numerous calibration metrics, and gate temperature for validation improvement, in order to reduce metric gaming. (5) Class imbalance/rarity. Long-Tail classes (such as rare varieties) will plague macro-F1 and ECE; for this reason, we also include confusion matrices and notes per class to put the errors in perspective (Fig 6):

## 6 Limitations and future work

**Frozen feature backbone.** However, our classifier only has fixed-size 1280-D embeddings and that does not reveal improvements in end-to-end processing and representation drift. Future: compare the feature space vs pixel space training (same split framework) Quantization/Pruning and how it affects the calibration

**Shifted-domain evaluation.** In this paper we show that noise-like OOD is a lower bound. Future: Multi-site/season/sensor folds, cross dataset transfer (Harmonized labella) AUROC for PE/BALD, E-AURC, selective prediction and Calibration (shifted) on test set (Sect 3.7). In addition, we currently assess ID vs OOD separation only under a synthetic, noise-like construction in feature space, which likely underestimates the difficulty of real shifts. Future work will therefore prioritize curated multi-season/multi-site splits and cross-device imaging scenarios to more rigorously validate shift awareness.

**Broader UQ and calibration baselines.** MC-Dropout and scalar temperature scaling are good, simple baselines. Future: deep ensembles, Laplace/Evidential/Dirichlet priors, isotonic/vector scaling, classwise prod temp, conformal prediction to give distribution-free coverage guarantees.

**Decision-centric evaluation.** Reliability should map to utility. Future: integrate explicit utility models (chemical cost, logistics, yield risk) and learn abstention thresholds on validation utility; report net-benefit or cost curves alongside standard metrics.

**Resource objectives and measurements.** The energy term is a clearly labeled, unitless proxy when power telemetry is unavailable. Future: anchor energy in Joules/inference on target devices, add memory footprint and throughput as explicit objectives, and report thermal throttling behavior. We also do not yet provide measurements on embedded hardware such as Jetson or smartphone-class ARM CPUs. Profiling on such devices, including memory footprint, thermal behavior, and battery impact under realistic flight or scouting missions, is an important next step for deployment-ready evaluation.

**Search budget and optimizers.** We used fixed-budget candidates to keep comparisons fair. Future: multi-fidelity schedules (Hyperband/BOHB), richer multi-objective solvers (NSGA-III, MOEA/D), and surrogate-assisted search to accelerate convergence without changing the evaluation protocol.

**Imbalance and rare classes.** Tail varieties/diseases remain challenging. Future: calibrated re-weighting, focal/cost-sensitive losses, targeted sampling, and per-class calibration auditing; couple these with active learning on high-uncertainty, low-support strata.

## 7 Conclusions

**Fig 10:**
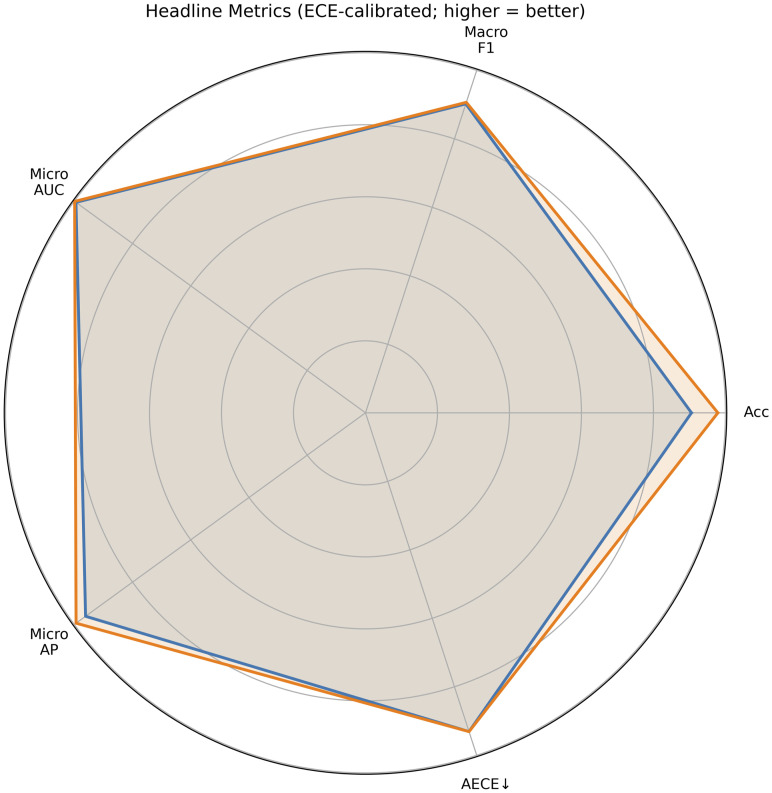
Headline radar plot comparing the disease and variety heads across Accuracy, Macro-F1, micro-AUC, micro-AP, and AECE. For readability, the AECE axis is inverted (larger is better after inversion). Values are from the *calibrated* state (Table 1/2): disease acc 0.906, macro-F1 0.902, micro-AUC 0.994, micro-AP 0.961, AECE 0.0138; variety acc 0.979, macro-F1 0.907, micro-AUC 0.999, micro-AP 0.994, AECE 0.0138.

We addressed rice disease and variety recognition through a *reliability-first, deployment-conditioned* approach that optimizes six objectives-error, calibration quality, uncertainty quality, size, latency, and an energy/runtime proxy-under a fixed evaluation budget. Concretely, we operate on compact 1,280-D embeddings with a two-head MTL classifier, estimate uncertainty via MC-Dropout, and apply *conditional* temperature scaling per head only when validation evidence warrants it. A solver-agnostic multi-objective search (in this instantiation) exposes the trade-space and selects a principled knee solution. As reported in Table 1 and Fig 10, the selected model couples strong discrimination with well-behaved probabilities and tight latency/size constraints-properties that matter directly for thresholded, on-farm decision making.

Methodologically, the contribution of the proposed MO-BBBC based framework is: (i) group-aware splits with leakage guards; (ii) uncertainty-aware training and selective-prediction reporting (risk–coverage and E-AURC); (iii) validation-gated calibration with full pre/post transparency on test; and (iv) Pareto/knee analysis with matched-budget baselines. This framework is *plug-compatible* with other HPO/MO solvers (NSGA-II/III, MOEA/D, BOHB) and with pixel-level backbones, making it easy to adopt in other crops and tasks (disease, pest, cultivar, severity).

Practically: calibrated probabilities allow us to use field thresholds that are local, indicating the costs of the field (chemical, travel, yield risk) without retraining, and the compact footprint and sub-millisecond latencies (Sect 4.7) suit handheld/UAV deployment. Reproducibility is ensured through a full trail of artifacts (including indexes for splits, saved imputers/scalers, checkpoints, auto-generated tables/figures, and in the form of:// JSON logs /ichter an, schempelnde Authentizitat), to support post-hoc auditing, as well as auto-scheduled re-calibration, which is increasingly common in some domain reviews. Using the same analysis toolbox, the same evaluation can be used to evaluate end-to-end backbones, telemetry-based energy (J/inference), richer UQ and calibration baselines (ensembles, Laplace/evidential, isotonic/vector scaling, conformal prediction), and real shifted-domain tests (site/season/sensor). We consider this a step forward in realizing agriculture vision systems with scores that mean what they say and with deployment constraints taken into consideration when selecting operating points.

### Contribution statement

In this work, **Chatter Singh** carried out the investigation and the evaluation of results. He also curated the dataset, as well as created visualizations to back up the findings. **Amar Singh** designed the study proposal, methodology, software, and formal analyses, overseeing the work and providing resources for the project. He was in charge of project administration and wrote the manuscript; both authors contributed to the writing, review, and editing of the manuscript. **Prof. Sahraoui Dhelim** provided conceptual guidance and supervision, contributed to study design refinements and critical revision of the manuscript, coordinated collaborations and resources, and served as the corresponding and submitting author.

### Data and code availability

All data underlying the findings in this study are available without restriction. The minimal dataset underlying the reported analyses (including the exact group-aware train/validation/test split indices, supporting metadata, and a complete reproducibility notebook) is publicly available on Zenodo at: https://doi.org/10.5281/zenodo.18471419.

The underlying images and labels used in this work come from the public PaddyDoctor image dataset [18], which can be accessed from the official project page https://paddydoc.github.io/dataset/ and via its IEEE DataPort record https://ieee-dataport.org/documents/paddy-doctor-visual-image-dataset-automated-paddy-disease-classification-and-benchmarking. The Zenodo record contains the files needed to reconstruct our exact experimental partitions from the original PaddyDoctor images and to reproduce the results reported in the manuscript.

All code used to implement the MO–BBBC framework, multitask classifier, uncertainty-aware curricula, calibration routines, and evaluation scripts (including leakage audits, OOD evaluation, and generation of all tables and figures) is freely available at: https://github.com/manhas82/MO-BBBC-Rice.git.

### Declaration of competing interest

The authors declare that they have no known competing financial interests or personal relationships that could have appeared to influence the work reported in this paper.

### Ethics statement

This study uses only publicly available plant imagery and does not involve human participants, animals, or any interventions requiring ethical approval.
